# Efficacy of a reduced-drug burden quadruple therapy with vonoprazan, cefuroxime, minocycline, and bismuth for *Helicobacter pylori* eradication: a retrospective study

**DOI:** 10.3389/fmed.2026.1897293

**Published:** 2026-08-03

**Authors:** Xuefeng Xu, Minghong Chen, Simin Chen

**Affiliations:** 1 Department of Gastroenterology, The Shengli Clinical Medical College, Fujian Medical University, Fuzhou, China; 2 Department of Gastroenterology, Fujian Provincial Hospital, Fuzhou, China; 3 Department of Gastroenterology, Fuzhou University Affiliated Provincial Hospital, Fuzhou, China; 4 Department of Orthodontics, Affiliated Stomatological Hospital of Nanchang University, Nanchang, China

**Keywords:** bismuth, cefuroxime, *Helicobacter pylori*, minocycline, quadruple therapy, vonoprazan

## Abstract

**Background and aims:**

Few studies have evaluated the efficacy and safety of vonoprazan, cefuroxime, minocycline, and bismuth (VCMB) quadruple therapy (with vonoprazan 20 mg once daily and cefuroxime 250 mg twice daily) for *Helicobacter pylori* (*H. pylori*) eradication. This retrospective pilot study aimed to assess the efficacy and safety of a 14-day VCMB regimen for *H. pylori* infection.

**Methods:**

This retrospective analysis enrolled patients treated with a 14-day VCMB quadruple therapy. The included patients were 18–80 years old and positive for ^1^^3^C urea breath test (^1^^3^C-UBT). VCMB quadruple therapy consists of vonoprazan 20 mg once daily, cefuroxime 250 mg twice daily, minocycline 100 mg twice daily and bismuth potassium citrate 220 mg twice daily. ^1^^3^C-UBT was repeated 4–6 weeks after treatment to evaluate the *H. pylori* eradication efficacy.

**Results:**

A total of 106 patients were enrolled. The intention to treat (ITT) and per-protocol (PP) eradication rates were 90.6% (95% CI: 82.9–95.1) and 96.0% (95% CI: 89.5–98.7). The overall incidence of adverse events was 21.9%. Mild dizziness and nausea were the most common adverse events, and these symptoms resolved spontaneously without additional treatment.

**Conclusions:**

The 14-day VCMB quadruple therapy, in which both vonoprazan and cefuroxime were administered at half their usual total daily doses, achieved a favorable *H. pylori* eradication rate, a low incidence of adverse events and good patient adherence. It is a potential new regimen for *H. pylori* eradication, further prospective multicenter studies are warranted to validate its efficacy and safety.

## 1 Introduction

*Helicobacter pylori* (*H. pylori*) infection is closely associated with gastric cancer (1), and eradication therapy can effectively reduce the incidence of gastric cancer (2, 3). In China, the national clinical guideline for *H. pylori* management recommends bismuth-containing quadruple therapy (BQT) as the preferred first-line treatment. Five antibiotic combination regimens are officially recommended in this guideline. Among them, four regimens contain amoxicillin, while the remaining one adopts a combined regimen of tetracycline and metronidazole (4). However, amoxicillin is not suitable for individuals allergic to penicillin, and such patients make up 5%−15% of the overall population (5), limiting its clinical utility. The use of conventional tetracycline is restricted by its limited availability in some regions and poor patient tolerance to the standard dosage of 500 mg three times daily. Additionally, the average *H. pylori* resistance rate to metronidazole in the Chinese population is as high as 75%−90% (6). Therefore, the clinical application of BQT is significantly limited by antibiotic resistance, penicillin-allergic and frequent adverse reactions. Although levofloxacin and clarithromycin are also recommended in the Chinese National Clinical Practice Guideline (4), their increasing resistance rates in recent years have impaired the success rate of *H. pylori* eradication. Thus, there is an urgent need to develop alternative antibiotic regimens that achieve high eradication rates while reducing adverse drug reactions and improving patient compliance.

Cephalosporins and amoxicillin belong to the class of β-lactam antibiotics and exert bactericidal effects on *H. pylori* via analogous pharmacological mechanisms (7). While approximately 10% cross-allergy reactivity has been documented between cephalosporins and amoxicillin, such allergic cross-reaction is clinically insignificant for second- and third-generation cephalosporins, and is largely confined to first-generation agents (8). Furthermore, previous studies have demonstrated that the *H. pylori* resistance rate to cefuroxime was comparable to that to amoxicillin (9, 10), supporting cefuroxime as a viable alternative to amoxicillin. The Maastricht VI Consensus Report and the updated domestic consensus guidelines for *H. pylori* eradication in China both recommend cefuroxime as an alternative option for patients with penicillin allergy (4, 11). A meta-analysis and literature summary conducted by Song et al. enrolled recent controlled studies on cefuroxime-based and amoxicillin-based anti-*H. pylori* regimens, and the results verified that the two regimens achieve similar eradication efficacy and exhibit comparable safety tolerability (12). This further validates cefuroxime as an alternative antibiotic for *H. pylori* eradication.

Minocycline is a semisynthetic tetracycline that binds to the 30S subunit of the bacterial 70S ribosome, thereby inhibiting peptide chain elongation and blocking bacterial protein synthesis (10). Minocycline exhibits higher lipid solubility, resulting in improved absorption and less interference from food intake (13, 14). Consistent with this, the Chinese National Clinical Practice Guideline has recommended minocycline as an alternative to tetracycline for *H. pylori* eradication (4).

In addition to rational antibiotic selection, sustaining gastric pH at around 6 during anti-*H. pylori* therapy is critical to guarantee eradication success and optimize treatment efficacy (15). Vonoprazan is a new oral potassium-competitive acid blocker (P-CAB), which binds competitively and reversibly to the potassium binding site of gastric proton pumps. Different from conventional PPIs, P-CABs act without the need for metabolic activation. They can rapidly raise intragastric pH within 4 h after initial administration and exert potent acid suppression lasting for 24 h. Furthermore, the acid-suppressive effect of vonoprazan is hardly influenced by diet status or CYP2C19 genetic polymorphisms, enabling more stable gastric acid regulation while maintaining a safety profile comparable to traditional PPIs (16).

BQT containing minocycline + amoxicillin or minocycline + metronidazole as first-line or second-line regimen for *H. pylori* eradication in patients not allergic to penicillin achieved satisfactory eradication efficacies, safety, and compliance (17). Based on the above analysis, vonoprazan, cefuroxime, minocycline, and bismuth (VCMB) quadruple therapy is expected to be a promising regimen for *H. pylori* eradication.

In our previous study involving tegoprazan—a P-CAB of the same class as vonoprazan—once-daily (QD) administration achieved an eradication rate comparable to that of twice-daily (BID) dosing (18). We suspected that vonoprazan 20 mg daily may be effective as a component of *H. pylori* eradication therapy. Therefore, vonoprazan was administered at 20 mg QD in the present study. Furthermore, although cefuroxime is most commonly used at 500 mg BID in previous reports, its optimal dosage in quadruple therapy remains unclear. Accordingly, we reduced the cefuroxime dosage to 250 mg BID in this study.

This study aims to retrospectively analyze the safety and efficacy of VCMB quadruple therapy in *H. pylori* eradication, to provide a basis for future research.

## 2 Method

### 2.1 Study design and participants

This was a single-center, retrospective observational study. We retrospectively screened patients receiving *H. pylori* eradication treatment from April 2025 to April 2026 through our hospital's outpatient electronic medical record system, and enrolled all eligible participants who satisfied the following inclusion criteria: (1) Patients aged 18 to 80 years with definite indications for *H. pylori* eradication therapy; (2) patients were treated with VCMB regimen. Exclusion criteria included patients: (1) Patients with a previous history of gastrointestinal surgery; (2) Individuals complicated with clinical conditions that might interfere with therapeutic evaluation, such as hepatic, renal or cardiac insufficiency, malignant tumors, and psychiatric disorders; (3) Patients who had taken proton pump inhibitors, antibiotics, anticoagulants or glucocorticoids within 8 weeks before enrollment, or required concurrent use of these medications during the treatment course.

### 2.2 Diagnosis and treatment of *H. pylori* infection

All enrolled patients were confirmed to have *H. pylori* infection via the ^1^^3^C urea breath test (^1^^3^C-UBT) (UCBT Kit; Atom High Tech, Beijing, China). Exhaled breath samples were collected 30 min after ingestion of the test reagent. A delta over baseline (DOB) value exceeding 4.0 was defined as a positive result, confirming *H. pylori* positive colonization. All patients were treated with a uniform 14-day quadruple eradication regimen, with the specific medication dosage and administration as follows: vonoprazan 20 mg, oral administration once daily, taken on an empty stomach in the morning; Cefuroxime axetil 250 mg, oral administration twice daily, taken 30 min after meals; Minocycline 100 mg, oral administration twice daily, taken 30 min after breakfast and dinner; Bismuth potassium citrate 220 mg, oral administration twice daily, taken 30 min before breakfast and dinner. The specifications and manufacturers of the study drugs were as follows: vonoprazan (20 mg per tablet, Takeda Pharmaceutical, Tianjin, China), minocycline (100 mg/tablet, Haichang Pharmaceutical Co., Ltd.), cefuroxime (cefuroxime axetil tablets 250 mg/tablet; Shenzhen Zhijun Pharmaceutical Co., Ltd.), bismuth potassium citrate (120 mg/tablet, Hunan Warner Pharmaceutical Co., Ltd.). All patients received a follow-up ^1^^3^C-UBT test 4–6 weeks after finishing the treatment course to evaluate the *H. pylori* eradication effect.

### 2.3 Data collection

Retrospective data were collected through the electronic medical record system of our hospital, including baseline information such as general demographic characteristics and treatment outcomes. Telephone follow-up was performed to record medication adherence, concomitant medications, and adverse events reported during treatment (such as nausea, diarrhea, abdominal pain, dizziness, rash, melena, etc.). The severity of adverse events was classified as mild, moderate, or severe, and corresponding management measures were documented. Symptom severity was assessed using a 0–10 scale (0 indicating no discomfort and 10 representing the most severe and intolerable symptoms). Good adherence was defined as a treatment completion rate of more than 80%.

### 2.4 Efficacy and safety evaluation

The primary outcome was the *H. pylori* eradication rate. Eradication rates were evaluated using intention to treat (ITT) and per-protocol (PP) analysis: ITT analysis, which included patients who received at least one dose of VCMB qual therapy, with those lost to follow-up or without outcome assessment regarded as treatment failures; PP analysis, which included patients who completed the full course of treatment, had good adherence (≥80%), and finished the ^13^C-UBT reexamination were included for analysis. Secondary outcomes included the incidence, type and severity of adverse events during treatment.

### 2.5 Statistical analysis

Data analysis was performed using SPSS 26.0 statistical software. Measurement data conforming to a normal distribution were expressed as mean ± standard deviation, while non-normally distributed data were presented as median (interquartile range). Categorical variables were expressed as numbers and percentages. The eradication rate was reported as percentage with its 95% confidence interval. The Wilson method with continuous correction was used to calculate 95% CI of the eradication rate.

### 2.6 Ethical review

This study protocol was approved by the Ethics Committee of our hospital (Approval No.: 202502202204000578660). As a retrospective study, it did not involve additional patient interventions, and the requirement for obtaining written informed consent was waived by the Ethics Committee. All patient data were anonymized to protect patient privacy.

## 3 Results

### 3.1 Baseline characteristics of enrolled patients

**Figure 1:**
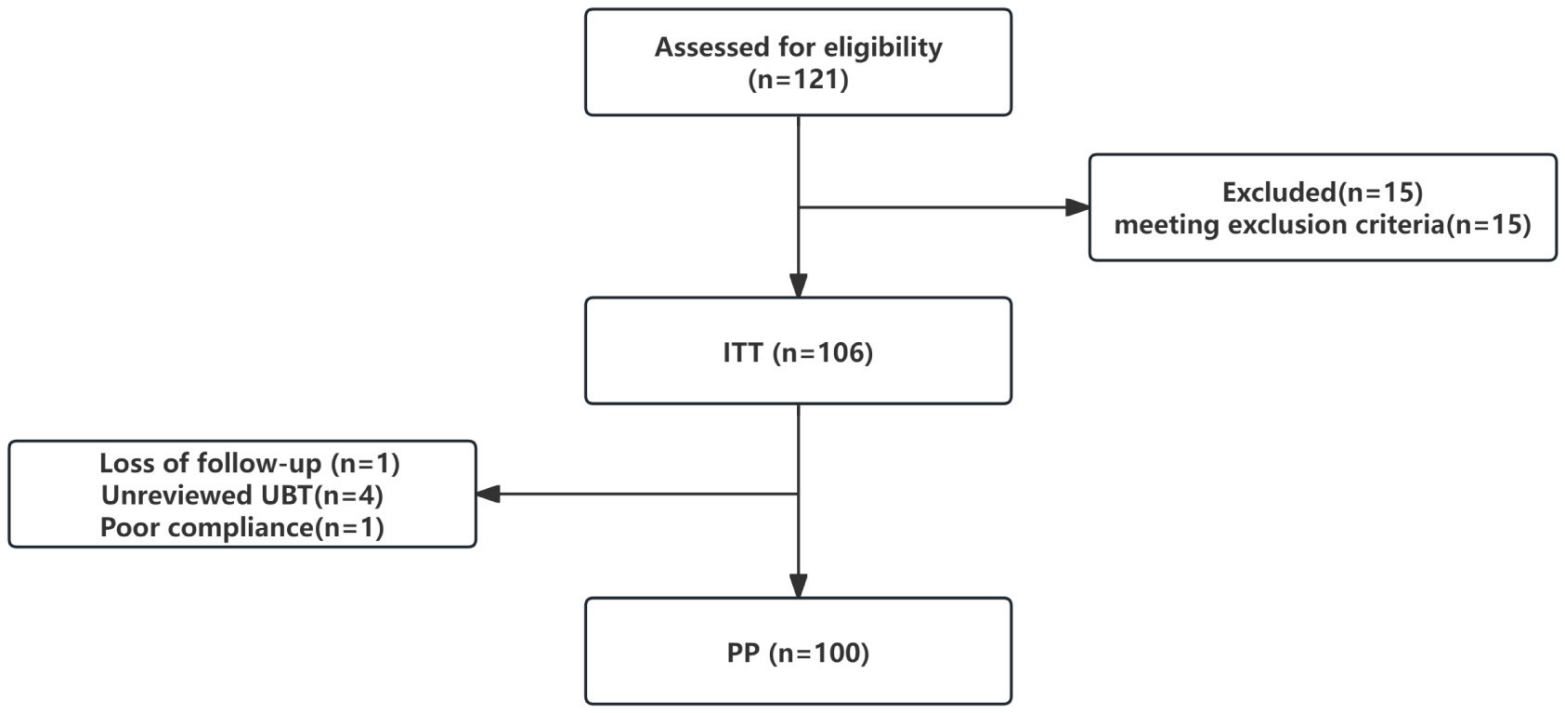
Flow chart of patient enrollment. ITT, intention-to-treat; PP, per-protocol; UBT, urea breath test.

A total of 121 patients who received the VCMB regimen for *H. pylori* eradication in the Gastroenterology Outpatient Department of our hospital from April 2025 to April 2026 were enrolled initially. After excluding 15 patients who failed to meet the inclusion criteria, a final total of 106 eligible patients were included in the study. The study flowchart is shown in Figure 1. The enrolled patients included 47 males (44.3%) and 59 females (55.7%). One patient was lost to follow-up, four patients did not undergo a subsequent ^13^C-UBT, and one patient had a treatment compliance rate below 80%. Among them, 89 patients were treatment-naïve, and 17 patients had at least one previous eradication attempt The demographic and clinical characteristics of the participants are summarized in Table 1.

**Table 1:** Baseline characteristics and demographic data of the included patients.

Characteristics	Total (*n* = 106)	Treatment naïve patients (*n* = 89)	Patients with one prior failure (*n* = 17)
Age, median years (IQR)	39 (30–51)	39 (30–54)	43 (33–47)
Sex (male/female)	47/59	39/50	8/9
BMI, median (IQR)	22.4 (20.3–24.2)	22.4 (20.3–24.3)	22.5 (20.8–23.9)
Cigarette smoking, *n* (%)	15 (14.2)	13 (14.6)	2(11.8)
Alcohol drinking, *n* (%)	13 (12.3)	9 (10.1)	4 (23.5)
Tea drinking, *n* (%)	32 (30.2)	23 (25.8)	9 (52.9)
Family history of gastric cancer, *n* (%)	6 (5.7)	4 (4.5)	2 (11.8)
Concomitant history			
Cardiovascular and cerebrovascular diseases (%)	3(2.8)	2(2.3)	1(5.9)
Respiratory diseases (%)	1(0.9)	1(1.1)	0
Chronic liver disease (%)	12 (11.3)	10 (11.2)	2 (11.8)
Chronic kidney disease (%)	0	0	0
Other diseases not affecting vital organs (%)	13 (12.3)	11 (12.4)	2 (11.8)
Gastroscopy diagnosis			
Peptic ulcer (%)	3 (2.8)	3 (3.4)	0
Non ulcerative disease (%)	26 (24.5)	24 (27.0)	2 (11.8)
Not available (%)	77 (72.6)	62 (69.7)	15 (88.2)

BMI, body mass index; IQR, interquartile range.

### 3.2 Eradication rates of *H.pylori*

The eradication rate of the VCMB regimen was shown in Table 2. Of the 106 enrolled patients, 96 achieved a negative ^1^^3^C-UBT result at 4–6 weeks after treatment completion, while 5 had treatment failure of *H. pylori* eradication. 1 patient was lost to follow-up, and 4 did not complete the re-examination. 1 patient had a treatment compliance rate below 80%. In the ITT analysis, eradication rate was 90.6% (95% CI: 82.9–95.1). In the PP analysis, eradication rate was 96.0% (95% CI: 89.5–98.7). Subgroup analysis by prior treatment history showed that among the 89 treatment-naïve patients, the ITT and PP eradication rates were 88.8% (79/89) and 95.2% (79/83), respectively, with an adverse event rate of 22.7% (20/88). In the 17 patients with one prior eradication failure, both ITT and PP eradication rates were 100.0% (17/17), and the adverse event rate was 17.6% (3/17). The eradication and adverse event outcomes for the initial treatment and retreatment subgroups are summarized in Table 3.

**Table 2:** Eradication rate of VCMB regimen.

Analysis	VCMB
ITT	90.6% (96/106)
95% CI	82.9–95.1
PP	96.0% (96/100)
95% CI	89.5–98.7

VCMB, vonoprazan 20 mg once daily, cefuroxime 250 mg twice daily, minocycline 100 mg twice daily and bismuth potassium citrate 220 mg twice daily; ITT, intention-to-treat; PP, per-protocol.

**Table 3:** Subgroup analysis of eradication and safety of VCMB quadruple therapy by prior treatment history.

Treatment times previously	ITT	PP	Adverse event rate
0	88.8% (79/89)	95.2% (79/83)	22.7% (20/88)
≥1	100.0% (17/17)	100.0% (17/17)	17.6% (3/17)

VCMB, vonoprazan 20 mg once daily, cefuroxime 250 mg twice daily, minocycline 100 mg twice daily and bismuth potassium citrate 220 mg twice daily; ITT, intention-to-treat; PP, per-protocol.

### 3.3 Adverse events and compliance

The adverse events of all patients are summarized in Table 4. In the examination of adverse events, 105 participants were included (excluding 1 lost to follow up). After 8 days of irregular medication, 1 patient discontinued treatment. A total of 23 patients (21.9%, 23/105) reported at least one adverse event, all of which were mild to moderate and did not require treatment interruption. The most common adverse reactions were nausea and dizziness, with an incidence of 4.8% for nausea and 9.5% for dizziness. No serious adverse events such as severe allergic reactions or hepatic and renal injury occurred. In terms of treatment compliance, 99.0% (104/105) of patients showed good compliance (>80%).

**Table 4:** Adverse events for VCMB regimen.

Adverse events	VCMB (*N* = 105)
Overall, *n* (%)	23 (21.9)
Abdominal pain, *n* (%)	1 (1.0)
Bloating, *n* (%)	0 (0.0)
Nausea, *n* (%)	5 (4.8)
Vomiting, *n* (%)	0 (0.0)
Constipation, *n* (%)	1 (1.0)
Belching, *n* (%)	0 (0.0)
Heartburn, *n* (%)	1 (1.0)
Diarrhea, *n* (%)	4 (3.8)
Abdominal discomfort, *n* (%)	0 (0.0)
Rash, *n* (%)	0 (0.0)
Dizziness, *n* (%)	10 (9.5)
Bitter taste, *n* (%)	0 (0.0)
Insomnia, *n* (%)	1 (1.0)
Anorexia, *n* (%)	0 (0.0)
Others, *n* (%)	0 (0.0)
AE grade, *n*	23
Mild	18 (78.3)
Moderate	5 (21.7)
Severe	0 (0.0)
Discontinued drugs because of AEs, *n*	0
Medication taken < 80%, *n*	1
Adherence, *n/N* (%)	104/105 (99.0%)

VCMB, vonoprazan 20 mg once daily, cefuroxime 250 mg twice daily, minocycline 100 mg twice daily and bismuth potassium citrate 220 mg twice daily.

## 4 Discussion

Our study is the first to explore the efficacy and safety of 14-day VCMB regimen for *H. pylori* infection. Overall, the PP and ITT eradication rates were 96.0 and 90.6%, respectively, with an adverse event rate of 21.9%. Subgroup analysis by prior treatment history showed that among the 89 treatment-naïve patients, the ITT and PP rates were 88.8 and 95.2%, respectively, while in the 17 patients with one prior failure, both rates reached 100.0%, with adverse event rates of 22.7 and 17.6%, respectively. The excellent efficacy observed in the prior-failure subgroup is clinically significant, especially considering that conventional rescue regimens such as levofloxacin-based triple therapy achieve only 82.0% (19). These findings suggest that VCMB may represent a valuable salvage option for patients with prior treatment failure. Nausea and dizziness were the most common symptoms, all of which were mild to moderate and resolved without additional intervention. In addition, patient adherence to this regimen reached 99.0%. Our findings indicate that VCMB therapy may be effective and well tolerated for *H. pylori* eradication.

BQT is currently the most widely recommended first-line regimen in both domestic and international guidelines. A multicenter randomized controlled trial published in 2024 (*n* = 1,300) reported PP and ITT eradication rates of 93.7 and 89.4%, respectively, for a 14-day BQT (20). Another randomized controlled trial focusing on clarithromycin-resistant patients reported ITT and PP eradication rates of 87.2 and 93.2%, respectively, for the same 14-day BQT (21). Vonoprazan-based BQT has also shown favorable efficacy; a meta-analysis of 10 studies reported ITT and PP eradication rates of 89.2 and 92.9%, respectively (22), while a retrospective study reported an eradication rate of 88.2% (23). In contrast, the traditional clarithromycin-based triple therapy, owing to escalating antimicrobial resistance, has seen its eradication rates decline to 62.0%−74.5% (24). Levofloxacin-based triple therapy is a commonly used second-line rescue regimen. A study reported eradication rates of 74.5%−80.9% for 14-day levofloxacin therapy (24), while the European Hp-EuReg registry demonstrated an overall second-line effectiveness of 82.0% (19). Vonoprazan-containing regimens have shown a pooled efficacy of 90% (87.0%−93.0%) in the rescue setting (25). Rifabutin-based triple therapy achieved eradication rates ranging from 80.7 to 100.0% in patients with multidrug-resistant infection (26). Our VCMB regimen achieved PP and ITT eradication rates of 96.0 and 90.6%, respectively, which are comparable to bismuth quadruple therapy, vonoprazan-based rescue, and rifabutin regimens, and superior to clarithromycin-and levofloxacin-based triple therapies.

Although vonoprazan-based regimens have shown advantages in efficacy and safety compared with traditional bismuth quadruple therapy, few studies have focused on the vonoprazan, cefuroxime, minocycline, and bismuth (VCMB) quadruple therapy, especially with the innovative modification of dose reduction. Notably, in this proposed VCMB regimen, both vonoprazan and cefuroxime are administered at half their usual total daily doses—a key innovation that differs from conventional full-dose regimens.

As a novel P-CAB, vonoprazan offers rapid action, sustained acid suppression, and insensitivity to CYP2C19 genetic polymorphisms. It confers better eradication potential than conventional proton pump inhibitors (PPIs) in *H. pylori* therapy (27–29). Although the standard vonoprazan regimen for *H. pylori* eradication is 20 mg twice daily, previous studies have demonstrated that vonoprazan 20 mg once daily achieves a comparable pH ≥4 holding time ratio (HTR), regardless of CYP2C19 genotypes, indicating non-inferior efficacy relative to esomeprazole 20 mg twice daily (28).The optimal dosage frequency of vonoprazan (20 mg twice daily vs. 20 mg once daily) in bismuth-containing quadruple therapy remains controversial. A 2026 prospective non-inferiority cohort study by Yan et al. compared the efficacy of these two regimens in *H. pylori* eradication (30). Among 201 enrolled *H. pylori*-positive patients, eradication rates were similar between the once-daily and twice-daily groups in both ITT (89.2 vs. 88.9%, *P* = 0.9) and PP analyses (92.2 vs. 95.2%, *P* = 0.4), verifying the non-inferiority of once-daily dosing. Furthermore, the once-daily regimen yielded a lower adverse event rate (8.9 vs. 12.0%) and superior cost-effectiveness (2.6 vs. 4.0). This high-level evidence supports the use of 20 mg once-daily vonoprazan in quadruple therapy. This simplified dosing strategy maintains eradication efficacy, improves safety, and facilitates treatment adherence in routine clinical practice.

Most prior studies administered cefuroxime 500 mg twice daily for *H. pylori* eradication. In a 2019 prospective cohort study involving 152 penicillin-allergic, treatment-naive patients, bismuth-containing quadruple therapy with cefuroxime, levofloxacin and esomeprazole achieved an ITT eradication rate of 85.5% (31). Another multicenter randomized controlled trial published in 2025 enrolled 248 comparable patients and verified a 90.32% ITT eradication rate using bismuth quadruple therapy combining standard-dose cefuroxime and tetracycline (32). Collectively, these data suggest that cefuroxime-based eradication efficacy varies with combination antibiotics, regional resistance patterns and patient profiles, and the optimal cefuroxime dosage in quadruple regimens remains unestablished. The current study adopted a modified quadruple strategy with cefuroxime axetil 250 mg twice daily, in combination with vonoprazan, minocycline and bismuth, and achieved favorable *H. pylori* eradication. This regimen offers considerable clinical value, particularly in settings of growing antibiotic resistance and unmet therapeutic needs for penicillin-allergic patients.

The favorable *H. pylori* eradication obtained with 250 mg twice-daily cefuroxime axetil in our study can be explained by two core mechanisms. Firstly, vonoprazan provides stable and potent gastric acid suppression. Since β-lactam antibiotics exert time-dependent bactericidal effects (33), elevated gastric pH promotes *H. pylori* growth and sensitizes bacteria to antibiotics, which compensates for the lower cefuroxime dosage. Secondly, cefuroxime and minocycline act synergistically via distinct antibacterial pathways. Minocycline targets the bacterial 30S ribosomal subunit, while cefuroxime acts on penicillin-binding proteins (34). Such mechanistic differences enhance bactericidal activity and eliminate the need for high-dose single antibiotics. A multicenter randomized trial by Zhang et al. confirmed the value of this combination (10). In 450 penicillin-allergic, treatment-naive patients, the 14-day bismuth quadruple therapy with standard-dose cefuroxime and minocycline achieved ITT and PP eradication rates of 82.7 and 90.9%, respectively. Its efficacy was comparable to metronidazole-based and furazolidone-based regimens, accompanied by a low adverse event rate of 22.6%. Only mild minocycline-related dizziness was observed, ensuring good safety and treatment adherence. Consistent with previous evidence, our study further confirms that reduced-dose cefuroxime (250 mg twice daily) achieves satisfactory eradication outcomes in vonoprazan-containing bismuth quadruple therapy with minocycline.

In terms of safety, adverse events in this study were consistent with those reported in other bismuth quadruple regimens, primarily including gastrointestinal discomfort and minocycline-related dizziness. In our study, the VCMB regimen was associated with an adverse event rate of 21.9%. Overall, adverse events were mild to moderate, mainly nausea and dizziness, and resolved spontaneously. Among first-line therapies, the reported adverse event rates for BQT vary considerably: a multicenter randomized controlled trial reported rates of 28.5% for 14-day BQT (20); a meta-analysis of vonoprazan-based BQT reported a rate of 37.3% (22). For rescue therapy, adverse event rates of BQT ranged from 35.1% to 41.7% across studies (35, 36), whereas high-dose dual therapy showed a significantly lower rate of 14.0% in a meta-analysis (35), and levofloxacin-based triple therapy was associated with a rate of 28.0% (19). The 21.9% rate observed in our regimen falls within the lower range of both first-line and rescue regimens. The excellent adherence rate of 99.0% indicated favorable tolerability and acceptability of this regimen. Clinically, the cefuroxime 250 mg twice daily and vonoprazan 20 mg once daily regimen provides improved safety and tolerability. Generally, antibiotic and acid-suppressant doses are positively correlated with adverse event rates. Gastrointestinal symptoms, such as diarrhea, bloating and nausea, commonly hinder the completion of *H. pylori* eradication therapy. As gastrointestinal upset is a typical adverse reaction of cefuroxime axetil, the 250 mg twice-daily regimen reduces single-dose drug exposure compared with the 500 mg twice-daily regimen, thereby improving patient adherence. Complete 14-day treatment courses are essential for successful eradication, and improved adherence is clinically critical for sustaining therapeutic efficacy.

There are several limitations in this study. First, although our study demonstrated favorable results, its generalizability is limited by the retrospective design, restrictive patient inclusion criteria and the relatively small sample size. Therefore, further well-designed randomized controlled trials are needed to validate the efficacy of this strategy in larger patient cohorts. Second, this study lacked a cefuroxime 500 mg twice-daily control, precluding direct efficacy comparison of the two doses. The observed dose optimization was supported by mechanistic evidence and indirect literature data. Moreover, the absence of susceptibility testing prevented accurate assessment of *H. pylori* cefuroxime resistance impacts on outcomes. Further susceptibility-guided comparative studies are needed to define the applicable populations and clinical settings for the 250 mg twice-daily regimen. Third, this study lacked complete data on patients' prior antibiotic use, making it impossible to evaluate the influence of prior cefuroxime and minocycline treatment on eradication outcomes.

## 5 Conclusion

Our retrospective study suggests that VCMB qual therapy may achieve a good eradication rate with a low incidence of adverse effects and high patient adherence. Although the doses of vonoprazan and cefuroxime in this regimen were half of the conventional standard doses (vonoprazan 20 mg once daily, cefuroxime 250 mg twice daily), this regimen maintained a satisfactory eradication rate while achieving a lower incidence of adverse reactions and higher treatment compliance. It demonstrates potential advantages in reducing medication burden and improving treatment tolerability for *H. pylori* eradication therapy. Large-scale, prospective randomized controlled studies should be conducted to further evaluate the efficacy and safety of VCMB qual therapy in *H. pylori* eradication.

## Data Availability

The original contributions presented in the study are included in the article/supplementary material, further inquiries can be directed to the corresponding author.
